# Supplementary material for: Spontaneous Vibrations and Stochastic Resonance of Short Oligomeric Springs
Source: Nanomaterials (Basel). 2023 Dec 22;14(1):41. doi: 10.3390/nano14010041 (PMC10780788; doi:10.3390/nano14010041)
Supplement: Supplementary file 1 [file nanomaterials-14-00041-s001.zip › nanomaterials-2757054-supplementary.pdf]

Supporting Information: Spontaneous Vibrations and Stochastic Resonance of Short Oligomeric Springs

Alexey M. Astakhov, Vladislav S. Petrovskii, Maria A. Frolkina, Anastasia A. Markina, Alexander D. Muratov,* Alexander F. Valov, and Vladik A. Avetisov*

N. N. Semenov Federal Research Center of Chemical Physics, Russian Academy of Sciences, Kosygina 4, 119991 Moscow, Russia

Design Center for Molecular Machines, Moscow, Russia

E-mail: alexander.muratov@chph.ras.ru; avetisov@chph.ras.ru

Simulation protocol

Parameters for Molecular Dynamics simulation

Morphology simulations were performed using the Gromacs2019 [S1] simulation package. Lennard–Jones parameters were taken from the OPLS-AA [S2] force field with a scaling factor of 0.5 for the 1–4 interactions. Long-range electrostatic interactions were treated using a smooth particle mesh Ewald technique [S3] with a cut-off of 1.2 nm. Bond vibrations were constrained with a LINCS [S4] algorithm. All calculations were performed in the NVT ensemble using the canonical velocity-rescaling thermostat, as implemented in the Gromacs2019 simulation package.

The parameterization of oligo-PP springs is presented in Figure S1a-b. The OPLS-AA

force field contains parameters only for single pyridine and furan molecules. First, $C4$ and $C6$ atoms have a covalent bond instead of hydrogen atoms and the partial charge of hydrogens were added to carbon atoms to maintain the neutrality. All the other partial charges are taken from OPLS-AA force-field. Second, there is only one undefined angle bond for atoms $C4 - C6 - N11$, which is $CW - CA - NC$ angle type in OPLS-AA. We used $CA - CA - NC$ angle due to similarity of CA and CW types. Both correspond to aromatic $sp2$-hybridized carbon.

The simulation was started from a random initial configuration. An equilibrated state were reached in two steps. First, a short run of 10 ps was carried out in the NVT ensemble with a time step of 0.01 fs with the leapfrog integrator for motion equations. Then the second part of equilibration was done for another 10 ns with a time step of 2 fs. After the equilibration, a long trajectory of 500 ns is obtained to achieve a clear picture of transitional behaviour.

To simulate the behaviour of the oligo-PP5 under the external load, one end of it was fixed and the other end was pulled by an external force (see Figure S1c). We used a simulation box of size $7 \times 7 \times 7\ nm^3$ due to technical aspects of Gromacs pulling algorithm. The axis of an initial conformation of oligo-PP5 was oriented in the XY plane. The longitudinal load F was applied along Z-direction to the center of mass of the last monomer unit and directed toward the attraction point, which was located along the vector connecting the left and right ends of the molecule.

In both cases, the center of mass of the first monomeric unit was fixed using a spring potential of $k = 100\,\mathrm{kJ \cdot mol^{-1} \cdot nm^{-2}}$; other specific constraints for bond length or atom positions were applied.

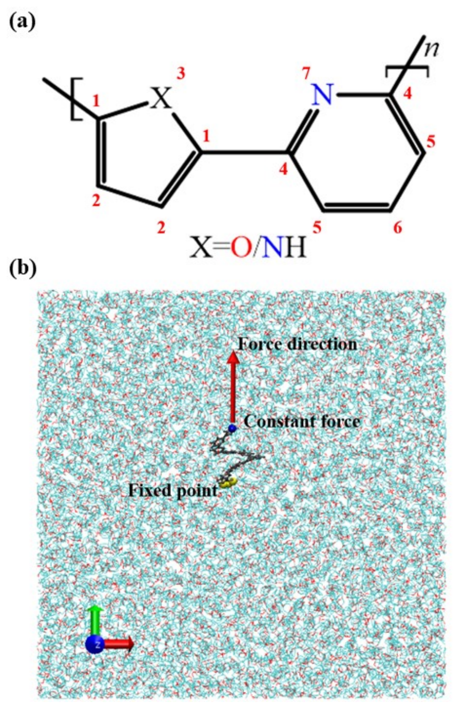

(c)

Atom number	OPLS-AA type	Partial charge
Cf1, Cp1	opls_567/543	+0.123/-0.008
Cf2, Cp2	opls_568/544	-0.154/-0.149
O3/N3	opls_566/542	-0.190/-0.239
C4	opls_521	+0.485
C5	opls_522	-0.447
C6	opls_523	+0.227
N7	opls_520	-0.678
H3	opls_545	+0.317
H5	opls_525	+0.155
H6	opls_526	+0.065
Hf2/Hp2	opls_570/547	+0.126/+0.118
H-term. pyridine	opls_524	+0.012
H-term. furan/pyrrole	opls_569/546	+0.142/+0.155

Figure S1: (a) The chemical structure of a PF/PP monomer, (b)a scheme of the simulation box for the oligo-PF5 system, (c) parameterization of PF/PP oligomers in OPLS-AA force field types and partial charges.

Oligo-PP-5 in water

In order to find out the impact of THF on the dynamics of oligo-PP, we also model its stretching in water. The simulation protocol in this case is similar to the one described in the section Parameters for Molecular Dynamics simulation above. One end of the oligo-PP5 was fixed and the pulling force was applied to another end. The end-to-end (R_e) distance both in water gradually increases along with the force up to the value of $F = 305\,\text{pN}$ at which point the oligo-PP5 helix turns to the fully stretched state. Note that the oligo-PP5 in water is present in the squeezed state at the pulling forces below $F = 305\,\text{pN}$ due to the hydrophilic nature of the solvent.

Parameters for periodic signal

Stochastic resonance was obtained by applying a periodic signal leading to the swinging of the oligo-PF/PP bistable potential. An oscillating force was implemented by setting a charge (-1) on the oligomer, adding a compensating charge ($+1$) as a counter ion in the solvent and applying a periodical electric field. The additional charge was placed on the end group of the oligo-PF-5/oligo-PP-5 and spread between the first, second and fifth atoms of the furan/pyrrole group (see Fig. S1a). The additional charges are equally distributed between the chosen atoms.

The periodic field in the Gromacs2019 package is defined by an equation

$$E(t) = E_0 \exp\left[-\frac{(t-t_0)^2}{2\sigma^2}\right] \cos\omega(t-t_0),$$

where the exponential part modulates the periodic part with the pulsing behaviour, E_0 is the amplitude of the signal and ωt_0 is the oscillation phase. Here we use only the static part when $\sigma = 0$ along y-axes (see Figure S1b). An external oscillating electrical field was directed along the constant force direction in the oligo-PF-5/oligo-PP-5 case.

Influece of the charges

The SR regime of the both oligo-PP-5 and oligo-PF-5 springs was examined by applying an additional oscillating electrical force on the unit charge preset on the pulling end of the spring while a compensative charge was placed $\sim 2.2\,\mathrm{nm}$ "below" the spring (if pulling direction is "up"). However, such a modification changed drastically the bistable behaviour of the springs: even with $\vec{F} = 0$ the bistability was observed with spontaneous vibrations present, while the equilibrium value of F_{eq} (when mean time spent in open and closed states is approximately equal) for oligo-PP-5 was only 3.5 pN. This result indicates that an artificial negative charge decreases the size of the closed state energy minimum, presumably because of its interactions with partial charges on the aromatic ring that comes in contact with the

charged end of the spring in closed state. We also observed that positively charged end of the spring influences the F_{eq} in opposite way (it increases compared to the non-charged case), which partially confirms the hypothesis about interactions with partial charges.

Torsion angle dynamics due to vibrations

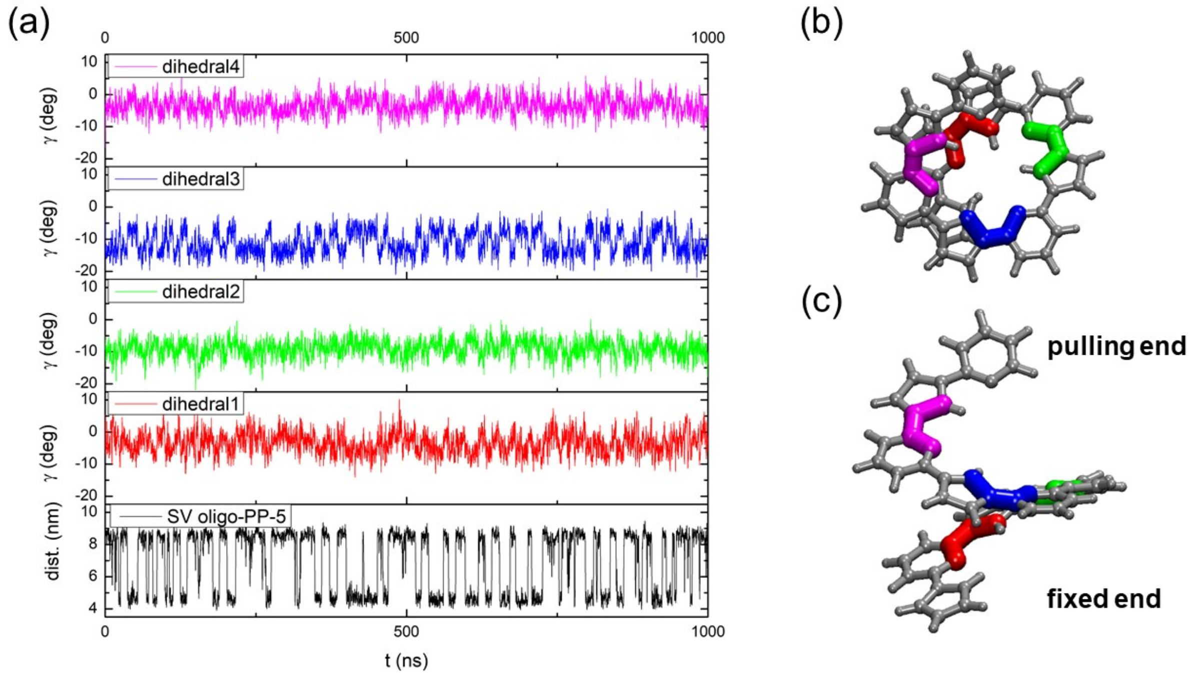

Figure S2: (a) Trajectories for the oligo-PP-5 spontaneous vibrations and N-C-C-N dihedrals along the spring structure. (b) Top and (c) side views of the oligo-PP-5 with selected dihedral groups.

We carried out a detailed analysis of torsion angles in the oligo-PP-5 spring during spontaneous vibrations. We chose the N-C-C-N dihedral as the most representative case since it connects different monomers. A distinguishable bistable trajectory is observed only for the dihedral, which bonds the 3rd and 4th monomers. The correlation coefficient for the trajectory of the dihedral torsion angle and the trajectory of oligo-PP-5 spontaneous vibrations was 0.86 (Figure S3a, blue curve). The other correlation coefficients had smaller values since the other monomers are closer to the oligo-PP-5 spring ends, which are under external restrictions such as fixing or force application.

Mean lifetime estimation

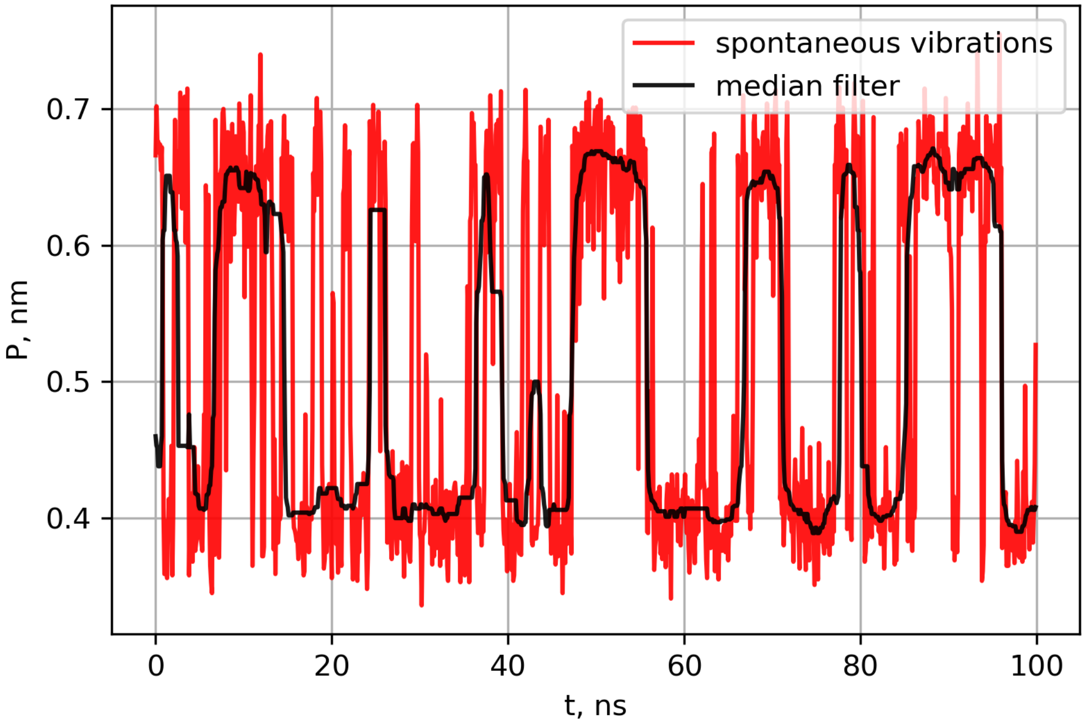

Figure S3: The spontaneous vibrations trajectory with and without the median filter usage.

In the oligo-PF/PP systems, one can observe the dynamics at two temporal scales: local and global. The first one is characterized by fast fluctuations near the squeezed/stress-strain states in case. However, the global dynamics (the switching between the ends) is more important for stochastic resonance. To calculate the mean lifetime, the local dynamics were excluded using the median filter $ndimage.median\_filter$ from an open-source software SciPy for Python 3 with a median filter window size of 3.5 ns, which corresponds to local vibrations. The resulting trajectory is shown in S3. According to the estimation, the mean lifetime for oligo-PF5 was $\tau = 6.14\,\text{ns}$ and corresponded to the external force $F = 279\,\text{pN}$.

References

(S1) Abraham, M. J.; Murtola, T.; Schulz, R.; Páll, S.; Smith, J. C.; Hess, B.; Lindahl, E. GROMACS: High performance molecular simulations through multi-level parallelism from laptops to supercomputers. *SoftwareX* **2015**, *1*, 19–25.

(S2) Kaminski, G. A.; Friesner, R. A.; Tirado-Rives, J.; Jorgensen, W. L. Evaluation and reparametrization of the OPLS-AA force field for proteins via comparison with accurate quantum chemical calculations on peptides. *The Journal of Physical Chemistry B* **2001**, *105*, 6474–6487.

(S3) Essmann, U.; Perera, L.; Berkowitz, M. L. A smooth particle mesh Ewald method. *The Journal of chemical physics* **1995**, *103*, 8577–8593.

(S4) Berk, H. P-LINCS: A parallel linear constraint solver for molecular simulation. *Journal of Chemical Theory and Computation* **2008**, *4*, 116–122.
